# Study on Anomalous Hall Effect and Spin–Orbit Torque Effect of TbCo-Based Multilayer Films

**DOI:** 10.3390/nano14090801

**Published:** 2024-05-05

**Authors:** Menglu Yang, Yuanjing Qu, Tao He, Xiong He, Yunli Xu, Lizhi Yi, Liqing Pan, Guangduo Lu

**Affiliations:** Hubei Engineering Research Center of Weak Magnetic-field Detection, College of Science, China Three Gorges University, Yichang 443002, China; ymlua47@163.com (M.Y.); 18203826687@163.com (Y.Q.); 18398932177@163.com (T.H.); hexiong@ctgu.edu.cn (X.H.); xuyunli_ctgu@163.com (Y.X.); lzhyi@ctgu.edu.cn (L.Y.); lpan@ctgu.edu.cn (L.P.)

**Keywords:** anomalous Hall effect, perpendicular magnetic anisotropy, spin–orbit torque effect

## Abstract

The anomalous Hall effect and spin–orbit torque of TbCo-based multilayer films have been methodically studied in recent years. Many properties of the films can be obtained by the anomalous Hall resistance loops of the samples. We report on the effects of a structure composed of two heavy metals as the buffer layers on the anomalous Hall resistance loops of TbCo-based multilayers at different temperatures. The results showed that the coercivity increases dramatically with decreasing temperature, and the samples without perpendicular magnetic anisotropy at room temperature showed perpendicular magnetic anisotropy at low temperatures. We quantified the spin–orbit torque efficiency and Dzyaloshinskii–Moriya interaction effective field size of the films W/Pt/TbCo/Pt at room temperature by measuring the loop shift of anomalous Hall resistance. The results showed that the study of anomalous Hall resistance loops plays an important role in the study of spintronics, which can not only show the basic properties of the sample, but can also obtain other information about the sample through the shift of the loops.

## 1. Introduction

The anomalous Hall effect (AHE) has always been the primary means of studying magnetic materials. The magnetic devices fabricated based on the anomalous Hall effect have the advantages of good thermal stability, a simple preparation process and film structure, low cost, etc., and do not require any external magnetic field. The magnetic properties of the samples can be known by measuring the current transport characteristics of the samples using AHE [1,2]. The AHE is one of the fundamental characterization tools for material systems at a given temperature, especially for perpendicular magnetized films [3]. The AHE curve can be used to judge whether the film has perpendicular magnetic anisotropy (PMA) [4]. The existence of PMA in Ta/CoFeB/MgO is proved experimentally by AHE curves [5]. PMA refers to the fact that when the ferromagnetic layer is very thin, the orbital anisotropy in the atom is reflected, and it is possible to make the interface anisotropy exceed the shape anisotropy, thus achieving PMA. This property can be applied to perpendicular magnetic anisotropy magnetic random access memory. Materials with a large AHE can be easily controlled for spintronic devices, which promotes the development of spintronic devices and magnetic random access memories (MRAMs) [6,7]. With the rapid development of the application of the AHE effect in thin-film materials, the phenomenon of the quantum anomalous Hall effect has been proposed. In 2013, the quantum anomalous Hall effect was observed experimentally for the first time. In 2017, jacal investigated the unconventional fractional quantum Hall effect in bilayer graphene [8]. In 2023, Chang et al. published another article, “Colloquium: Quantum anomalous Hall effect” [9]. Research on the anomalous Hall effect is ongoing. Furthermore, when PMA films are used as the materials of spin–orbit torque magnetic random access memories (SOT-MRAMs), higher memory density and more potent magnetic moment flipping stability can be obtained [10,11,12,13].

One of the main problems faced by the application of SOT to the technical field is the conversion efficiency and thermal stability of charge flow and spin flow. Rare earth (RE)–transition metal (TM) alloys have the outstanding advantages of tunable physical parameters (magnetic moments, etc.) and perpendicular magnetic anisotropy (PMA) of bulk materials, which are candidates for the next generation of spintronics and ideal carriers for the study of SOT information for functional materials and devices. These materials provide an internal antiferromagnetic coupling and avoid difficulties such as the lack of compelling read and write mechanisms in antiferromagnetic materials [14,15]. The RE-TM amorphous films with PMA were prepared firstly through the sputtering method by Gambino et al. in 1973 by using GdCo [16]. Since then, much research has been conducted on RE-TM alloy films. Strong SOT efficiency was found in RE-TM alloy films, which is mainly reflected near the component compensation point, where there is a divergence trend [17]. TbCo is a well-known RE-TM alloy with many excellent properties, especially when the Tb atomic percentage is about 25% [18]. In addition, studies have shown that when the Tb/Co layer is thinner, its magnetic properties behave like the TbCo alloys [19]. In 2021, Zheng et al. proposed a field-free DMI-SOT switching scheme in a thick perpendicular CoTb layer with a vertical composition gradient [20]. The intrinsic quality of TbCo itself also has an impact on the whole sample, and single-layer TbCo can also produce SOT efficiency and have superior properties [21,22].

At present, the research on spin–orbit moment (SOT) efficiency mainly focuses on two aspects: One is to study how to achieve deterministic magnetization flip under zero magnetic field and to no longer need external auxiliary fields to break the symmetry of the system, which is of great significance for the wide application of SOT-MRAMs. How to use the SOT effect and all-electric manipulation method to achieve the magnetic moment flip of the vertical free layer has become one of the critical issues in promoting the industrialization of SOT-MRAM devices in the world. The second is how to accurately quantify the magnitude of the equivalent field of damping and the equivalent field of the quasi-field term, as quantitatively measuring the magnitude of the effective field of the spin–orbit moment is an important basis for the in-depth study of the internal mechanism of the spin–orbit moment, improving the efficiency of the spin–orbit moment. Characterization of SOT in the PMA system is of great importance for further study of the internal mechanism and screening of regulatory film materials. In addition to the Second Harmonic Hall Voltage method [23], measurement of spin torque ferromagnetic resonance [24] and magneto-optical Kerr effect measurements [25], a simple current-assisted domain wall (DW) propagation model was proposed by Pai et al. in 2016 [26]. Based on measuring the AHE, this simple measurement scheme allows SOT efficiency to be quantified and simultaneously provides an estimate of the chiral DMI effective field [27,28,29].

In this study, we focused on studying the anomalous Hall resistance loops of Tb_29_Co_71_-based multilayer films at different temperatures. The coercivity of all samples increases with the decrease in temperature, and the samples without PMA showed PMA at low temperatures. Meanwhile, the SOT efficiency and DMI effective field size of W/Pt/TbCo/Pt at room temperature were measured through our experimental test platform. The measurement results show that the anomalous Hall resistance loops can be shifted under a smaller longitudinal in-plane bias field.

## 2. Materials and Methods

**Figure 1:**
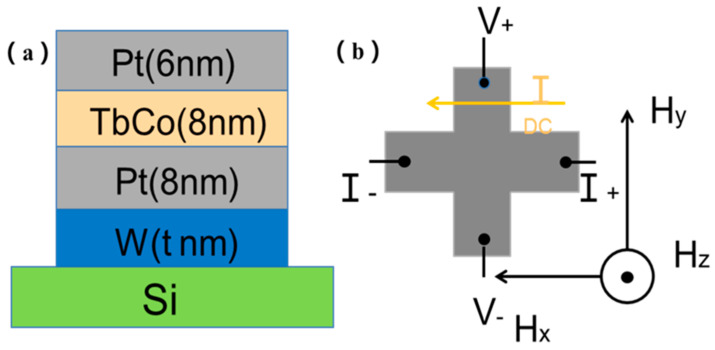
(**a**) A schematic diagram of the W/Pt/TbCo/Pt film heterostructure deposited on the Si substrate. (**b**) An illustration of the device structure with the measurement set-up. The directions of DC flow (I_DC_), the out-of-plane applied field (H_z_), the longitudinal field along the wire (H_x_), and the transverse field (H_y_) are shown.

The W(t)Pt(6)/TbCo(8)/Pt(6) films (thickness in nm) were prepared by radio frequency magnetron sputtering (Chinese Academy of Sciences Shenyang Scientific Instrument Development Center, Shenyang, China) at room temperature, and the thickness of W ranged from 0 nm to 10 nm. A 6 nm layer of Pt was deposited on top of the TbCo layer to protect the films from oxidization. All of the films were deposited on a Si (100) substrate. As shown in the Figure 1a, the Tb_29_Co_71_ (at. %) films were deposited at 100 W. The sputtering power of W and Pt was kept at 30 W. The base pressure of the chamber was less than 2 × 10^−4^ Pa. The Ar working gas pressure was 0.4 Pa. We used optical lithography and ion milling to pattern multilayers into the Hall bar structures (Institute of Optoelectronic Technology, Chinese Academy of Sciences, Sichuan, China), where the current channel was 10 μm (width) × 150 μm (length), and the voltage channel was 10 μm (width) × 75 μm (length). Figure 1b illustrates the measurement configuration. We obtained the results of the AHE curve and SOT efficiency at room temperature using a self-built test platform, in which currents were generated by using Keithley 6221 and voltages were measured by using Keithley 2182. The AHE at low temperature was measured in a VersaLab system by using Quantum Design.

## 3. Results

**Figure 2:**
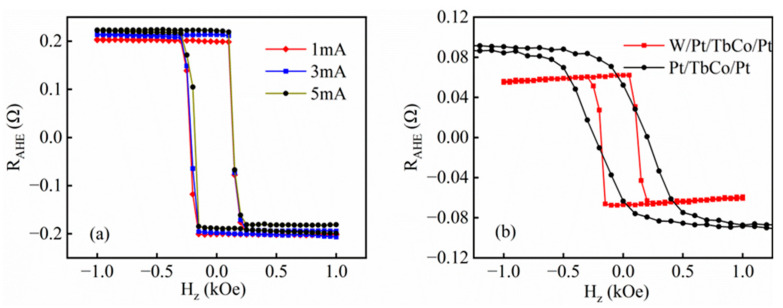
(**a**) Anomalous Hall resistance loops of W(1)/Pt(8)/TbCo(8)/Pt(6) (thickness in nm) measured at different currents at room temperature. (**b**) Anomalous Hall resistance loops of W(3)/Pt(8)/TbCo(8)/Pt(6) and Pt(8)/TbCo(8)/Pt(6).

In order to verify the performance of the sample, the anomalous Hall loop of the sample was measured through a self-built measurement platform, and the vertical anisotropy of the sample was verified through the anomalous Hall loop. We first discuss the results of the AHE at room temperature using a self-built test platform. The anomalous Hall resistance loop of W(1)/Pt(8)/TbCo(8)/Pt(6) (thickness in nm) is measured at different currents, as Figure 2a shows. After processing and calculation, the same sample is measured at different currents, and it is found that the loops of the sample do not change. It proves that the sample was less affected by Joule heat and had commendable thermal stability [30]. The measurement of multiple samples reveals the same properties, which shows that the influence of ambient temperature on the test system is eliminated and verifies the stability and reliability of the test platform that we built.

Figure 2b shows the anomalous resistance loops of W(3)/Pt(8)/TbCo(8)/Pt(6) and Pt(8)/TbCo(8)/Pt(6). It can be seen from the figure that W(3)/Pt(8)/TbCo(8)/Pt(6) has better PMA at room temperature. One of the possible explanations for these results is that the structure consisting of two heavy metals as buffer layers has caused other effects from the additional W/Pt interface. The effect of this structure on SOT was studied by Ma et al. in 2018 [31]. A novel switching mechanism was demonstrated via two spin currents of opposite spin indices by Ma. The competing spin currents generate an effective SOT with an effective perpendicular field that can switch a PMA layer without any applied magnetic field. In order to further verify the role of a bilayer heavy metal structure in the preparation of thin films, the anomalous resistance loops with different W and TbCo thicknesses were measured at room temperature, and all samples with W layers showed commendable PMA. Therefore, all our subsequent studies have adopted this structure.

**Figure 3:**
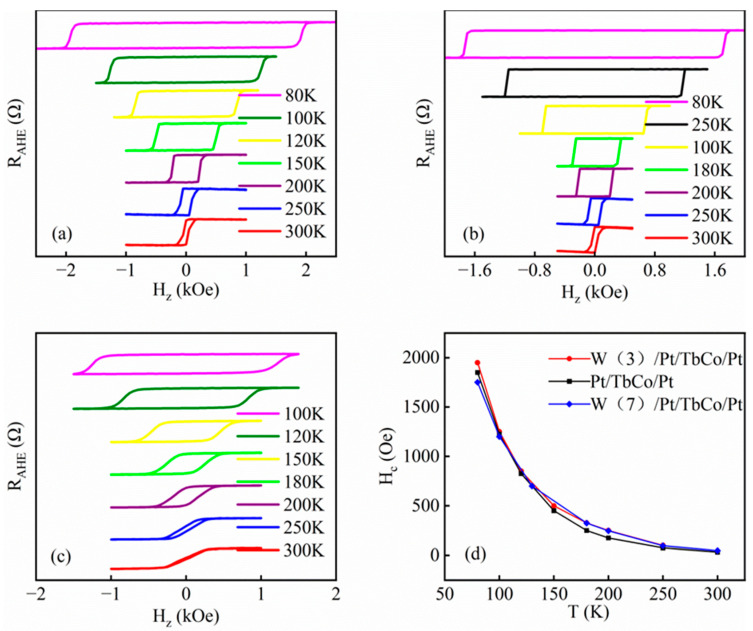
(**a**–**c**) Anomalous Hall resistance loops at temperatures from 80 K to 300 K for W(3)/Pt(8)/TbCo(8)/Pt(6), W(7)/Pt(8)/TbCo(8)/Pt(6) and Pt(8)/TbCo(8)/Pt(6), respectively. The resistances are not drawn to scale. (**d**) The variation in the coercive force of samples with temperature.

In general, materials and devices may operate in a wide temperature range, and it is important to study the effect of temperature on their performance. In order to meet the requirements of electronic devices for different temperatures, we study the effects of temperatures on the anomalous Hall resistance loops in the following. The anomalous resistance loops of W(3)/Pt(8)/TbCo(8)/Pt(6), W(7)/Pt(8)/TbCo(8)/Pt(6) and Pt(8)/TbCo(8)/Pt(6) were measured by VersaLab system at a temperature from 80 K to 300 K, as shown in Figure 3a–c. Figure 3a,b show the anomalous resistance loops of W(3)/Pt(8)/TbCo(8)/Pt(6) and W(7)/Pt(8)/TbCo(8)/Pt(6) from 80 K to 300 K. The results show that the samples of W/Pt/TbCo/Pt have excellent PMA at both room temperature and low temperature; meanwhile, with the decrease in temperature, the coercivity increases clearly. Figure 3c shows the anomalous resistance loops of Pt(8)/TbCo(8)/Pt(6) from 100 K to 300 K. Judging from the previous test results, we learned that Pt/TbCo/Pt has no PMA at room temperature. However, it can be seen from our test diagram that with a temperature decrease, the PMA of the films reappears. The results show that the coercivity of all samples increases with the decrease in temperature, as shown in Figure 3d.

Low temperature can affect the coercivity and other properties of the sample [32]. Our experiments confirm this concept. In the meantime, by measuring the anomalous Hall resistance loops of the Pt/TbCo/Pt samples at different temperatures, it was found that the low temperature also affected the PMA of the samples. This applies mainly to samples that do not exhibit PMA at room temperature. The experiment proves that the sample whose performance is not superior at room temperature may be more suitable for use at low temperatures. At temperatures below 150 K, the Pt/TbCo/Pt samples without PMA at room temperature showed renewed PMA. This experimental phenomenon is consistent with the findings of study on interlayer coupling of samples at low temperatures by Liu et al. conducted in 2012 [33]. The study indicates that the interlayer coupling between the prepared nanowires increases rapidly when the temperature drops from 300 K to 100 K, and then changes slightly. However, for the continuous film with PMA, the interlayer coupling is almost independent of temperature. This is consistent with our experimental phenomenon. Our experimental research found that the multilayer film with PMA at room temperature does not change much at low temperatures. In contrast, the sample without PMA at room temperature exhibited renewed PMA at low temperatures, mostly around 150 K. It is suggested that the reason for the reappearance of PMA at low temperatures may be related to the enhancement of interlayer coupling caused by magnetostatic interaction between stray fields.

**Figure 4:**
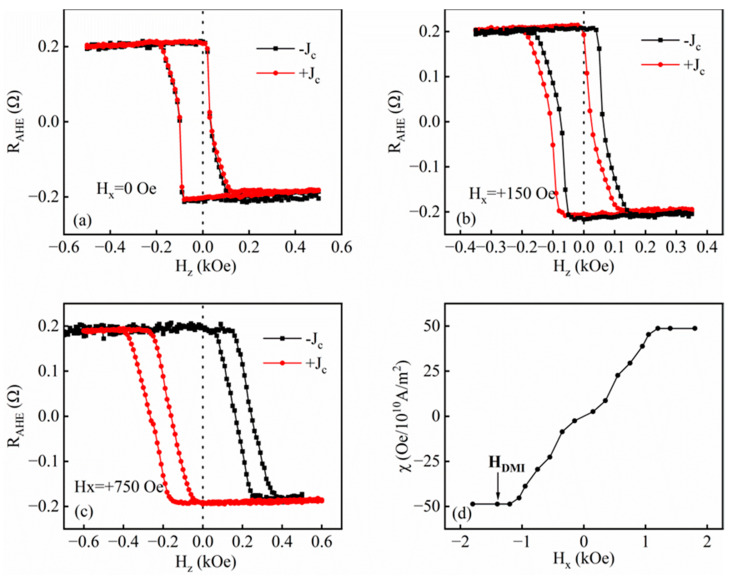
(**a**–**c**) Anomalous resistance loops of the samples of W(3)/Pt(8)/TbCo(8)/Pt(6) under I_DC_ without and with a longitudinal in-plane bias field of H_x_ = 150 Oe and H_x_ = 750 Oe at room temperature, respectively. (**d**) The spin–orbit torque efficiency (χ) as a function of H_x_.

Based on the properties of the sample obtained above, we further studied the SOT of the films W(3)/Pt(8)/TbCo(8)/Pt(6) at room temperature using the self-built testing platform, with currents generated by using Keithley 6221 and voltages measured via Keithley 2182. A magnet with an adjustable magnetic field size was added along the x-direction of the test platform to change the size of the in-plane bias field in the test. Then, the current was introduced to continue to measure the anomalous Hall curve of the sample, and the measured curve was offset at this time. The offset of the curve gradually increased and tended to be saturated with the increase in the size of the bias field, and the SOT efficiency of the sample and the size of the DMI effective field were measured by this method. Figure 4a–c show the anomalous Hall resistance loops for the sample of W(3)/Pt(8)/TbCo(8)/Pt(6) under I_DC_ without and with a longitudinal in-plane bias field of H_x_ = 150 Oe and H_x_ = 750 Oe, respectively. An applied in-plane bias field is used to break the homochiral Néel DW of samples. At this time, in the case of an incoming current, the effective field generated by the current exerts the same direction on the vertical components of the two DWs, and the torque makes the domain walls move in two opposite directions, which will lead to the expansion of the magnetic plate and eventually the reversal. As can be seen, when the current polarity is changed without an in-plane bias field, the anomalous resistance curves of the sample completely overlap and do not shift. When an in-plane bias field of 150 Oe is applied, the curve shifts, currents of different polarity pass through, and the curve shifts in the opposite direction.

As the in-plane bias field increases, the average deviation in the loop line also increases, and the chirality of the DWs is wholly destroyed and finally reaches saturation. Figure 4d shows the slope of H_z_ per unit current density. This slope, which relates to the SOT efficiency χ, was measured as a function of the longitudinal and transverse bias fields. The calculation formula of SOT efficiency is χ = H_z_^eff^/J_c_. In the case of applying a magnetic field, χ varies linearly with H_z_ and saturates above a threshold. We note that when applying a transverse field of at least 1200 Oe, we observe neither a loop shift nor a change in coercivity, indicating that the size of the DMI effective field is about 1200 Oe. In the saturated field, the SOT efficiency is χ = 48.71 Oe/10^10^A·m^−2^ according to calculations. The temperature also affects the spin–orbit torque of the sample. In 2018, Pham et al. highlighted the importance of studying thermal contributions in SOT switch experiments by studying the effect of temperature on magnetization reversal [34]. It can be expected that the SOT efficiency and the DMI effective field size of samples at low temperatures will be better because the samples have better PMA at low temperatures, as shown in Figure 3. These results are useful for further research on electrical transport in the RE-TM alloy material system. However, due to the limitation of experimental conditions, we are unable to obtain the data at low temperatures at present; this needs to be further studied in the future.

## 4. Conclusions

In conclusion, we characterized the effects of a structure composed of two heavy metals as the buffer layers on the anomalous Hall resistance loops of TbCo-based multilayer films at different temperatures. We found that different buffer layers greatly influence the PMA of the samples at room temperature. The coercivity increases dramatically and enhances as the temperature drops, and the samples without PMA at room temperature showed PMA at low temperatures. We attribute this to the improved interlayer coupling and magnetic anisotropy energy increases of the samples at low temperatures. Then, we measured and calculated the SOT efficiency and DMI effective field size of W/Pt/TbCo/Pt at room temperature. We have obtained decent efficiency in the sample. The DMI effective field size of the sample is about 1200 Oe, and the SOT efficiency in the saturated field is χ = 48.71 Oe/10^10^A·m^−2^. These results of our study are helpful to stimulate the further study of the AHE and SOT in RE-TM alloy materials and provide an experimental basis for the RE-TM alloy thin-film material system to become the next generation of new storage materials.

## Data Availability

The data presented in this study are available on request from the corresponding author.
